# Correction to “Pthirus pubis infestation of the scalp in a 4‐month‐old infant:A case report”

**DOI:** 10.1111/srt.70337

**Published:** 2026-02-13

R. Zhu, S. Huang, D. Yang, X. Yang, L. Peng, L. Zhou, X. Qi, L. Ren, and M. Guo, “Pthirus Pubis Infestation of the Scalp in a 4‑Month‑Old Infant: A Case Report,” *Skin Research and Technology* 29, no. 3 (2023): e13299, https://doi.org/10.1111/srt.13299.

The “Ethics Statement” section as originally published was inappropriate, as it included the full text of the informed consent signed by the participant's legal guardian and identifiable information. The “Ethics Statement” has been corrected in the published article as follows:

This study was retrospectively approved by the Ethics Committee of People's Hospital Of Leshan, adhering to the Declaration of Helsinki and relevant Chinese ethical guidelines.

The“ Patient Consent” section is also inaccurate. The “Patient Consent” section should read as follows:

The patient's parents have provided informed consent for the publication of the case.

The authors apologize for these errors and for the inconvenience they may have caused.

